# From Triumph to Trial: A Case Study of Non-Tuberculous Mycobacterium After Tetralogy of Fallot (TOF) Correction in an Immunocompetent Child

**DOI:** 10.7759/cureus.62207

**Published:** 2024-06-11

**Authors:** Salman Khan, Yamna Ali, Khalid Saifullah Baig, Ujala Hussain, Ziad Ali

**Affiliations:** 1 Cardiovascular Diseases, Rehman Medical Institute, Peshawar, PAK; 2 Internal Medicine, Hayatabad Medical Complex Peshawar, Peshawar, PAK; 3 Internal Medicine, Khyber Teaching Hospital, Peshawar, PAK; 4 Internal Medicine, Rehman Medical Institute, Peshawar, PAK

**Keywords:** mycobacterium simiae, tuberculosis, nontuberculous mycobacteria, tetralogy of fallot, mycobacterium tuberculosis, child

## Abstract

*Mycobacterium simiae*, a slow-growing non-tuberculous mycobacterium (NTM), presents diagnostic challenges due to its resemblance to *Mycobacterium tuberculosis *and other NTMs. While NTM infections and tuberculosis share clinical and radiological features, their management strategies differ. Accurate differentiation is pivotal, as misdiagnosing NTM infections such as MDR-TB can lead to ineffective treatments. A case involving an 11-year-old female with tetralogy of Fallot (TOF) and a pulmonary *M. simiae* infection underscores the importance of precise diagnosis. Enhancing diagnostic methods is imperative to prevent mismanagement of NTM infections and ensure appropriate care.

## Introduction

Nontuberculous mycobacteria (NTMs) refer to all individuals of the genus *Mycobacterium* that are not part of the *Mycobacterium tuberculosis* complex (MTBC) or* Mycobacterium leprae* [1]. One of the NTMs with the slowest growth rate, *Mycobacterium simiae*, was first discovered in rhesus monkeys in 1965 [2]. The prevalence of NTM disease has been variably estimated across distinct geographic regions [3]. NTM diseases exhibit clinical and radiological presentations similar to tuberculosis. The risk variables common to both tuberculous and NTM patients include advanced age, immunosuppression, organ transplantation, HIV infection, structural cardiac disease, and underlying pulmonary illness. Both types of organisms are naturally acid-fast [4]. *Mycobacterium avium* complex (MAC) is by far the most typical organism causing pulmonary illnesses among the NTMs. *Mycobacterium kansasii* and *M. abscessus* are some other causative entities [5]. The rare cause of lung infections caused by NTMs, however, is* M. simiae*. We report a case of pulmonary infection caused by *M. simiae* in a young female with underlying structural heart diseases, i.e., tetralogy of Fallot (TOF).

## Case presentation

An 11-year-old female postoperative for a Blalock-Thomas-Taussig shunt for TOF started to have complaints of cough with little mucoid sputum, a high-grade intermittent fever, a loss of appetite, and weight loss of about 1.2 kg. Between the surgery and the onset of symptoms, there was a seven-day window. On examination, the patient was well-oriented to time, place, and person. There was no evidence of icterus, clubbing, lymphadenopathy, cyanosis, pallor, or pedal edema. Her vital signs were as follows: breathing 30 bpm, blood pressure 120/80 mmHg, and maintaining oxygen saturation 98% with 4 liter oxygen provided via a face mask. Bilateral normal vesicular breath sounds were audible with moderate ejection systolic murmur during chest auscultation.

The full blood picture revealed a leukocyte count of 7600 cells per mm^3^ with a normal differential count and a hemoglobin level of 12 gm%. Renal and liver function tests were normal. Viral screening was negative for SARS-CoV-2, and the pleural fluid sample was exudative.

**Figure 1:**
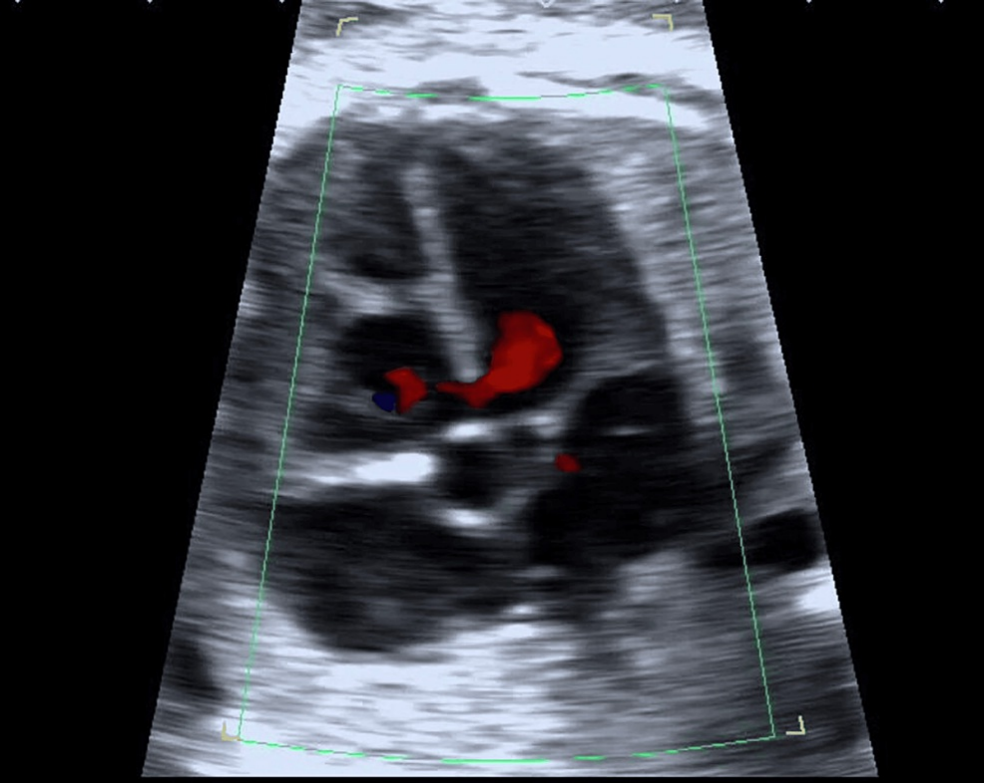
Echocardiogram showing ventricular septal defect (VSD)

**Figure 2:**
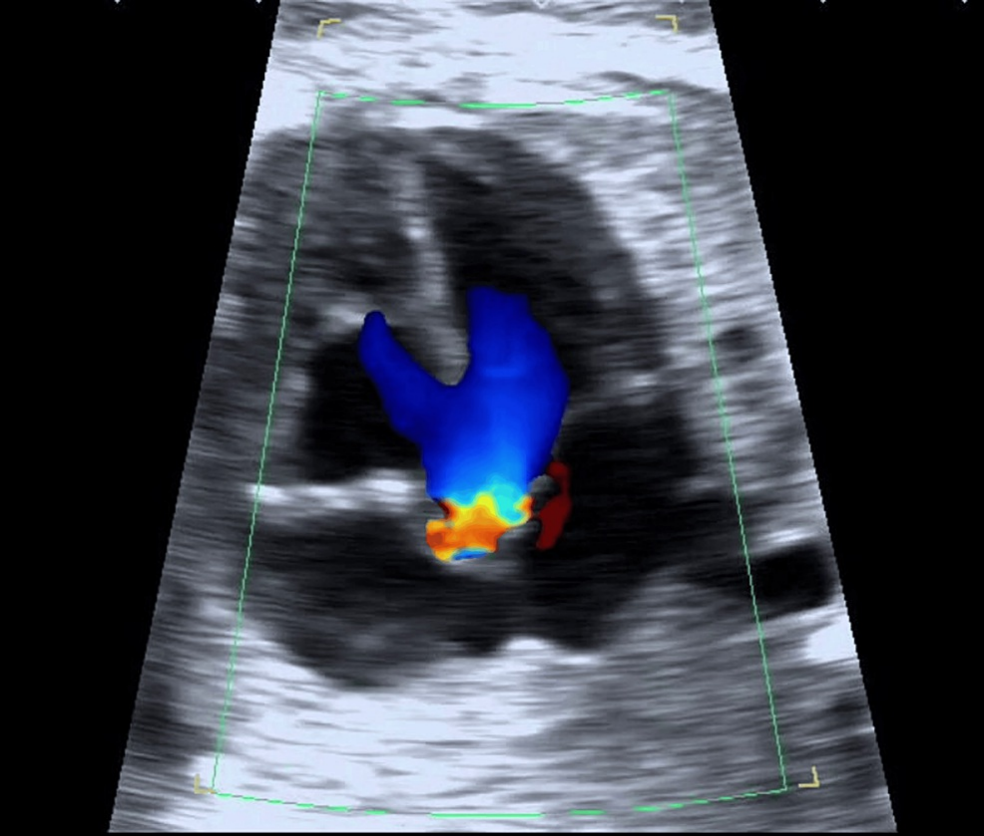
Echocardiogram suggesting overriding of the aorta

**Figure 3:**
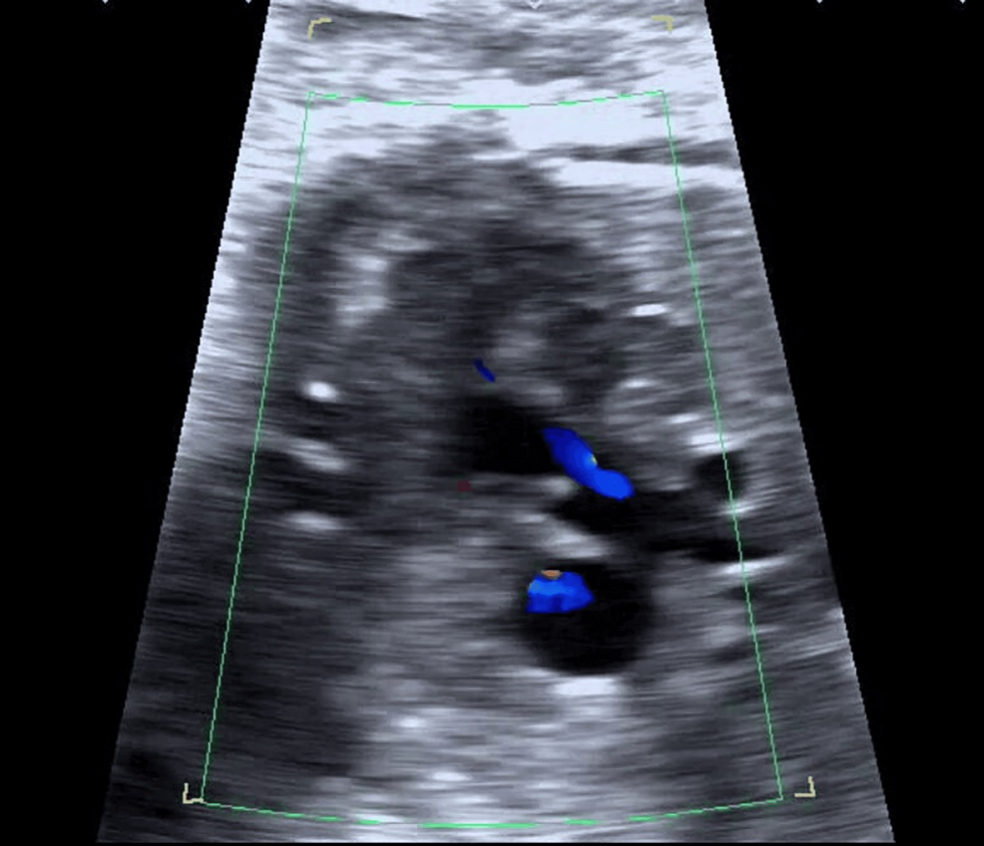
Echocardiogram showing severe pulmonary hypoplasia

**Figure 4:**
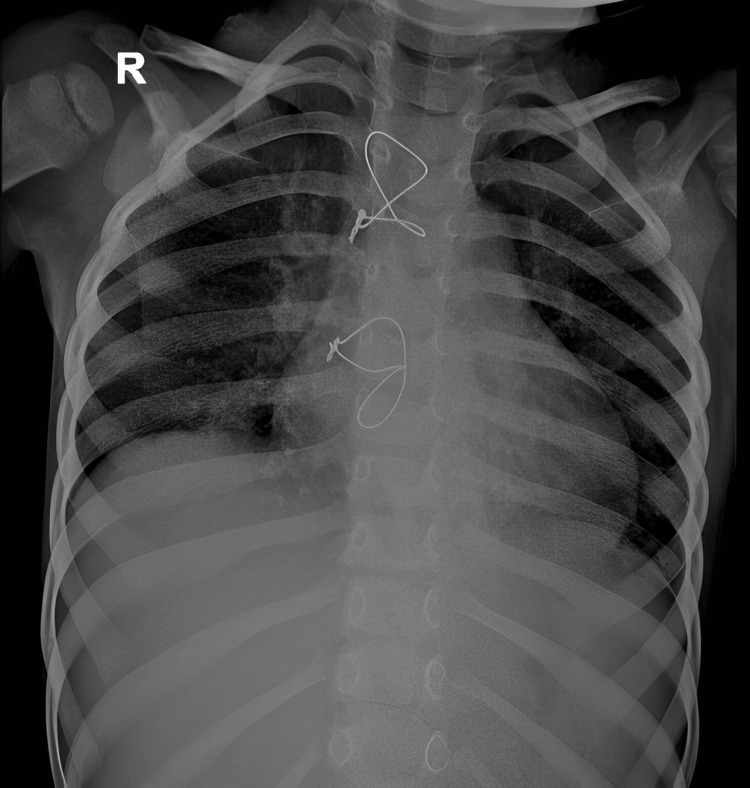
Chest X-ray demonstrating post sternotomy and bilateral hilar opacities with mild cardiomegaly

The preoperative echocardiogram (Figures 1-3) revealed a ventricular septal defect (VSD), overriding of the aorta, and pulmonary hypoplasia. Chest radiograph (Figure 4) showed bilateral hilar opacities with mild cardiomegaly, and computed tomography revealed bilateral gross pleural effusion.

The Mantoux test was 5 mm, but acid-fast bacilli (AFB) were not found in the sputum analysis. The bronchial washes showed AFB, but Gene-Xpert failed to find MTB. Based on the results, the possibility of NTMs was considered, and a sample was sent for mycobacterial culture. After four weeks, the Lowenstein-Jensen medium culture showed the growth of an NTM, and *M. simiae *was determined to be the species by DNA sequencing. Based on the culture and sensitivity results, the patient was started on intravenous amikacin. After six weeks, the patient improved objectively and subjectively, and she was not producing sputum either.

The patient was discharged on the same treatment regimen and advised to continue the treatment for three months until the completion of therapy. Upon follow-up, another culture was taken, which yielded no microbial growth.

## Discussion

Small, rod-shaped bacilli belonging to the Mycobacteria family can be divided into two primary categories, NTM and MTBC, which includes the strains of *tuberculosis*, *bovis*, and *africanum*. Although more than 150 different NTM species have been identified, *Mycobacterium abscessus*, *Mycobacterium kansasii*, and MAC are the most frequent causes of lung infections [6]. Since NTM infections closely resemble other chronic or subacute infections, cancers, and autoimmune disorders clinically and radiographically, a high degree of suspicion is required to make the diagnosis of NTM. A study showed that many identified cases of TB in Brazil were NTM cases, and patients were given the incorrect combination of drugs with numerous potential side effects.NTM pulmonary disease (NTM-PD) has frequently been confused with multidrug-resistant tuberculosis (MDR-TB) in locations with a high TB prevalence [7]. When exposed to light, *M. simiae* is a photochromogen, which creates a yellow-orange pigment. In 1965, *M. simiae *was first isolated from rhesus macaque monkeys [8].

Similar to other NTMs, *M. simiae* can cause disseminated infections, lung illnesses, lymphadenitis, and skin and soft tissue disorders, especially in AIDS patients. An immunocompetent patient developed a disseminated *M. simiae *infection, as reported by Maher et al. *M. simiae*-related pulmonary symptoms are non-specific. Cough, sputum production, hemoptysis, sweating, weight loss, low-grade fever, and dyspnea are common presenting symptoms of infected patients [9]. Chest X-ray, if done, in the early stages of the disease, can be nonspecific and normal [10]. Most often, the infection affects older people and those with diabetes mellitus, AIDS, cardiovascular diseases, chronic lung diseases, and cancers [11,12]. In one case series, the average age was 61 years. However, some case reports also mention *M. simiae*-related infections in children [13]. Sputum smear microscopy is the main technique used to diagnose tuberculosis in many impoverished nations. An NTM infection is frequently misinterpreted as tuberculosis because Ziehl-Neelsen (ZN) staining cannot distinguish between mycobacterial species and the initial laboratory diagnosis is frequently "smear-positive." As a result, anti-tuberculosis therapy is given to the majority of patients with an NTM lung infection [14]. It has been observed in Iran that patients with NTM infections who do not respond to anti-TB medication may be mistakenly diagnosed as having MDR-TB [7]. Due to the small number of case reports and series, no conclusive information regarding the ideal regimen and length of treatment is available. Because *M. simiae* is the NTM with the highest drug resistance, medical treatment alone has a very low success rate [15]. We initially began treating our patient using oral azithromycin along with intravenous amikacin and imipenem for six weeks during which she showed clinical and radiologically improvement. She was discharged under the same regime to be continued for three months. A recent retrospective analysis recommended clarithromycin for a term of six to 24 months in various combinations with cotrimoxazole, moxifloxacin, and amikacin [12].

## Conclusions

In low-resource healthcare facilities, the availability of diagnostic services for *Mycobacterium tuberculosis* culture and speciation is inadequate. To address this gap, it is of vital importance to make progress in enhancing culture and speciation services across all levels of healthcare. This improvement is crucial to facilitate the diagnosis of NTM diseases accurately and to provide tailored treatment options, potentially yielding more favorable outcomes.
